# Comparative analysis of genetically-modified crops: Part 1. Conditional difference testing with a given genetic background

**DOI:** 10.1371/journal.pone.0210747

**Published:** 2019-01-16

**Authors:** Changjian Jiang, Chen Meng, Adam Schapaugh

**Affiliations:** Global Regulatory Sciences, Monsanto Company, Chesterfield, Missouri, United States of America; Huazhong University of Science and Technology, CHINA

## Abstract

The European Food Safety Authority (EFSA) mandates two sets of statistical tests in the comparative assessment of a genetically-modified (GM) crop: difference testing to demonstrate whether the GM crop is different from its appropriate non-traited control; and equivalence testing to demonstrate whether it is equivalent to conventional references with an history-of-safe-use. The equivalence testing method prescribed by EFSA confounds the so-called GM trait effect with genotypic differences between the reference varieties and non-traited control. Critically, these genotypic differences, which we define as a ‘control background effect’, are the result of conventional plant breeding. Thus, the result of EFSA equivalence testing often has little or nothing to do with the GM trait effect, which should be the sole focus of the comparative assessment. Here, an integrated method is introduced for both difference and equivalence testing that considers the differences of the three genotype groups (GM, control, and references) as a two-dimensional random variable. A novel statistical model is proposed, called the trait model, that treats the effects of the GM and control materials as fixed for their difference, and as random for their common background. For significance testing, the covariance structure of the three genotype groups is utilized to decompose the differences into the trait effect and the control background effect. The trait difference is then derived as a conditional mean, given the background effect. The comparative assessment can then focus on the conditional mean difference, which is independent of the control background effect. Furthermore, the trait model is flexible enough to include various types of genotype-by-environment (G×E) interactions inherent to the experimental design of the trial. Numerical evaluations and simulations show that this new method is substantially more efficient than the current EFSA method in reducing both Type I and Type II errors (protecting both the consumer and producer risk) after the background effect is removed from the test statistic, and successfully addresses two major criticisms (i.e. statistical model lack of G×E, and study-specific equivalence criterion) that have been raised.

## Introduction

In the European Union, the safety of a genetically-modified (GM) crop and derived food/feed is established, in part, using a proof-of-equivalence approach [1]. Under this paradigm, any phenotypic or compositional differences between the GM crop and its near-isogenic, non-traited comparator are evaluated in the context of natural variation, estimated from conventionally-bred varieties (hereafter, references) grown in the same field trials. Currently, the GMO Panel of the European Food Safety Authority (EFSA) mandates two sets of statistical tests in the comparative assessment of a GM crop [2]: difference testing to demonstrate whether the GM crop is different from its near-isogenic, non-traited comparator; and equivalence testing to demonstrate whether it is equivalent to conventional references with an history-of-safe-use. Per [2], this assessment should be based on data from a minimum of eight sites in one growing season (or two growing seasons each with four sites). Within each site, three genotype groups are assigned to plots under a randomized complete block design. The genotype groups include: a test (GM) variety, a control (the near-isogenic comparator), and a set of (minimum six) conventional references.

At least three linear mixed models have been proposed for this design ([3] (this paper presents the method in [2]), [4, 5]). All three models include a fixed factor ‘genotype group’, which distinguishes the test, control, and group of references as a whole, and a fixed or random factor ‘genotype’ with as many levels as there are materials. Differences between the three genotype groups can be driven by one or more mechanisms. Because the genetic composition of the test and control are very similar, any difference between the two is a potential ‘trait effect’ and becomes a focus of the comparative assessment [6, 7]. Differences between the control and references, although not directly considered by [2], are part of the natural variation inherent to the crop; these differences are defined as a ‘control background effect’. Critically, differences between the test and references can be driven by a trait effect, a control background effect, or both. However, although the control itself is needed, the control background effect is not relevant in terms of the comparative assessment because differences between the control and references are the result of conventional plant breeding, and do not involve the trait.

**Fig 1:**
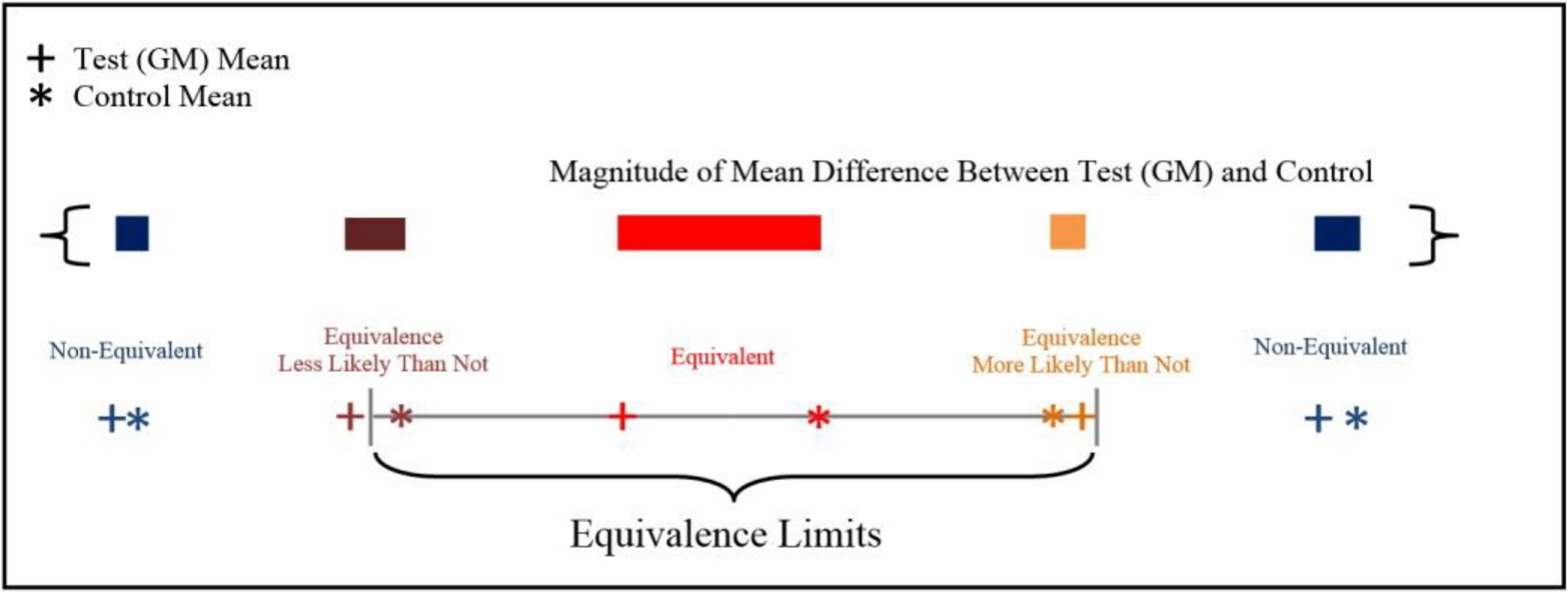
Graphical illustration of EFSA equivalence category in relation to the background effect. The background effects are genotypic differences between reference varieties and the conventional control, a result of conventional plant breeding. Conclusions of EFSA equivalence are often driven by the background effect and have little or nothing to do with a GM trait. In this hypothetical (but very realistic) example, the test with the largest trait effect (i.e. largest mean difference, shown in red) is the only one deemed to be equivalent.

EFSA’s method [2] for equivalence testing assesses differences between the test and references. These differences, as described above, may be driven by a trait effect, a control background effect, or both. In all three linear mixed models noted above, however, the trait effect and control background effect are confounded, and cannot be decomposed. Thus, the result of EFSA equivalence testing often has little or nothing to do with the trait effect (Fig 1), which should be the sole focus of the comparative assessment. To address this problem, an integrated method is introduced for both difference and equivalence testing that considers the differences of the three genotype groups as a two-dimensional random variable. A novel statistical model is proposed, called the trait model, that treats the effects of the test and control materials as fixed for their difference, and as random for their common background. For significance testing, the covariance structure of the three genotype groups is utilized to decompose the differences into the trait effect and the control background effect. The trait difference is then derived as a conditional mean, given the background effect. The comparative assessment can then focus on the conditional mean difference, which is independent of the control background effect. The trait model is also flexible enough to separate the various types of genotype-by-environment (G×E) interactions, one for each type of genetic effect, inherent given the design of the experiment. Numerical evaluations and simulations show that this new method is substantially more efficient than the current EFSA method [2] in reducing both Type I and Type II errors, and successfully addresses two major criticisms of EFSA that have been raised (i.e., the inability to model G×E interactions; and study-specific equivalence criteria, making the GM trait much less relevant than the control in the equivalence testing [4]). The paper is organized as follows: the derivation of the conditional method and introduction of the trait model are presented first, followed by an application using empirical field data. Results of numerical evaluations and simulations are presented alongside a re-analysis of a maize grain composition example by the conditional method. The paper concludes with a discussion of the trait model as it compares to the EFSA method [2], additional thoughts on G × E interactions, and highlights areas for further research.

## Materials and methods

### The TCR trial

The Test-Control-Reference (TCR) trial is the experimental base of EFSA’s prescribed two-step difference and equivalence testing methodology. Despite differences in the number of sites, reference varieties, etc., all TCR trials share some common properties. Let (Δ_*TC*_, Δ_*CR*_, Δ_*TR*_) denote genotype group differences of the test versus control, control versus references, and test versus references, respectively. Let (*D*_*TC*_, *D*_*CR*_, *D*_*TR*_) be the sample estimates and $\sigma_{D_{TC}}^{2},\;\sigma_{D_{TR}}^{2},\;\sigma_{D_{CR}}^{2},\;\sigma_{D_{TC},D_{CR}},\;\sigma_{D_{TC},D_{TR}}$, and $\sigma_{D_{TR},D_{CR}}$ be the respective variances and co-variances. Under common statistical assumptions, i.e. independent and identically-distributed responses, two basic properties of all TCR trials are:

- Genetic relationship: Both Δ_*TC*_ and Δ_*TR*_ contain the trait effect, but Δ_*TR*_ also contains Δ_*CR*_ due to Δ_*TR*_ = Δ_*TC*_ + Δ_*CR*_, and Δ_*CR*_ represents the control background effect;
- Experimental design: The paired arrangement of the test and control materials in the field, along with the above genetic relationship, leads to the following relationships:

$$\sigma_{D_{CR}}^{2}=\sigma_{D_{TR}}^{2},\;\sigma_{D_{TC},D_{CR}}=-\sigma_{D_{TC},D_{TR}}=-\frac{1}{2}\sigma_{D_{TC}}^{2}\leq 0,\;\sigma_{D_{TR},D_{CR}}=\sigma_{D_{CR}}^{2}-\frac{1}{2}\sigma_{D_{TC}}^{2}\geq 0.$$

Notice in property [a] that the differences of the three genotype groups are two dimensional: one dimension is the control background effect; the other dimension is the GM trait effect. While both Δ_*TC*_ and Δ_*TR*_ are measures of the GM trait effect, in principle, only one dimension should be left for the comparative evaluation of the trait. Conceptually, a new parameter, possibly a combination of Δ_*TC*_ and Δ_*TR*_ (since each one cannot represent the other), must exist for the best measure of the GM trait difference, without confounding by the background effect.

Property [b] is a direct result of the field arrangement. For example, $\sigma_{D_{CR}}^{2}$ and $\sigma_{D_{TR}}^{2}$ are the sampling variations of *D*_*CR*_ and *D*_*TR*_, respectively, and should be equal due to the paired arrangement in the field and the common background effect. Similarly, the covariance of *D*_*TC*_ with *D*_*TR*_ should be the same as with *D*_*CR*_. Other relationships can also be derived among variances and covariances.

The control material is the result of conventional plant breeding and there is no obvious preference for one control over other commercially-available varieties. Thus, the control background effect should be considered as random, and its potential value is no different from any individual reference in the reference genotype group. The random control background has been considered by [2], [5], and [8] in establishing the equivalence criterion, but not properly modeled (as will be shown in the following trait model) and incorporated in the statistical testing. In this investigation, this has been an underlying assumption in every step of the analysis, including the development of hypotheses, estimation of the trait effect, and the derivation of test statistics.

The remainder of this section addresses three fundamental questions: (1) how do we define the GM trait effect for a given background; (2) how do we model the random background effect in addition to other factors; (3) what is the sample performance of the estimation procedure?

### GM trait difference for a given control background

Given Δ_*TR*_, a mixture of the GM trait- and background effects can be decomposed using conditioning or regression. Under the assumption of a bivariate normal distribution of (*D*_*TC*_,*D*_*CR*_), consider the following hypothetical model:

$$\left\{ \begin{array}{l} D_{TC}=\alpha +\beta D_{CR}+\varepsilon \\ D_{TR}=\alpha +(1+\beta {)D}_{CR}+\varepsilon \end{array}\right.$$

Let $\mu_{D_{TC}|D_{CR}},\;\mu_{D_{TR}|D_{CR}},\;\sigma_{D_{TC}|D_{CR}}^{2}$, and $\sigma_{D_{TR}|D_{CR}}^{2}$ denote the conditional mean and variance of *D*_*TC*_ and *D*_*TR*_ given *D*_*CR*_. Using the notation of conditioning or regression, the model can be specified as: $\alpha =\mu_{D_{TC}|D_{CR}=0}=\mu_{D_{TR}|D_{CR}=0},\;\sigma_{\varepsilon }^{2}=\sigma_{D_{TC}|D_{CR}}^{2}=\sigma_{D_{TR}|D_{CR}}^{2}$, and *β* depends on the correlation. The two equations in this model are linearly related, thus only one model equation is necessary since *D*_*TR*_ = *D*_*TC*_ + *D*_*CR*_. This implies that, given *D*_*CR*_, evaluation based on both *D*_*TC*_ and *D*_*TR*_ would be the same, and can be replaced by the evaluation of the intercept $\alpha =\mu_{D_{TC}|D_{CR}=0}=\mu_{D_{TR}|D_{CR}=0}$. The application of the above model depends on the correlation structure among the three genotype groups, which was derived in the last section. The objective of the conditioning is to eliminate the dependence of Δ_*TR*_ (when its estimate is applied in the equivalence testing) on Δ_*CR*_ (the control background effect). This is achieved by making use of the estimated background effect.

Let $\rho_{D_{TC},D_{CR}}$ denote the correlation coefficient between *D*_*TC*_ and *D*_*CR*_. The following equations are direct applications of bivariate normal theory [9]:

$$\left\{ \begin{array}{l} \mu_{D_{TC}|D_{CR}}=E[D_{TC}|D_{CR}]=\Delta_{TC}+\rho_{D_{TC},D_{CR}}∙\frac{\sigma_{D_{TC}}}{\sigma_{D_{CR}}}∙(D_{CR}-\Delta_{CR})\\ \sigma_{D_{TC}|D_{CR}}^{2}{=\sigma }_{D_{TC}}^{2}(1-\rho_{D_{TC},D_{CR}}^{2}) \end{array}\right.$$ (1)

$$\left\{ \begin{array}{l} \mu_{D_{TR}|D_{CR}}=E[D_{TR}|D_{CR}]=E[(D_{TC}+D_{CR})|D_{CR}]=\mu_{D_{TC}|D_{CR}}+D_{CR}\\ \sigma_{D_{TR}|D_{CR}}^{2}=\sigma_{(D_{TC}+D_{CR})|D_{CR}}^{2}=\sigma_{D_{TR}|D_{CR}}^{2} \end{array}\right.$$ (2)

where $\rho_{D_{TC},D_{CR}}=\frac{\sigma_{D_{TC},D_{CR}}}{\sigma_{D_{TC}}\sigma_{D_{CR}}}$. Substituting the corresponding equations in property [b], (1) and (2) become:

$$\left\{ \begin{array}{l} \mu_{D_{TC}|D_{CR}}=\Delta_{TC}-\frac{\sigma_{D_{TC}}^{2}}{{2\sigma }_{D_{CR}}^{2}}(D_{CR}-\Delta_{CR})\\ \sigma_{D_{TC}|D_{CR}}^{2}{=\sigma }_{D_{TC}}^{2}(1-\frac{\sigma_{D_{TC}}^{2}}{4\sigma_{D_{CR}}^{2}}) \end{array}\right.$$ (3)

$$\left\{ \begin{array}{l} \mu_{D_{TR}|D_{CR}}=\Delta_{TC}+D_{CR}-\frac{\sigma_{D_{TC}}^{2}}{{2\sigma }_{D_{CR}}^{2}}(D_{CR}-\Delta_{CR})\\ \sigma_{D_{TR}|D_{CR}}^{2}=\sigma_{D_{TC}}^{2}(1-\frac{\sigma_{D_{TC}}^{2}}{{4\sigma }_{D_{CR}}^{2}}) \end{array}\right.$$ (4)

By conditioning on the estimated background effect, we do not obtain total independence of Δ_*CR*_ in $\mu_{D_{TR}|D_{CR}}$. However, $\mu_{D_{TR}|D_{CR}}$ in (4) becomes only fractionally-dependent on Δ_*CR*_, and $\sigma_{D_{TR}|D_{CR}}^{2}$ is even less than $\sigma_{D_{TC}}^{2}$, which is known to be free of background variation.

Also, notice that the expectations of $\mu_{D_{TC}|D_{CR}}$ and $\mu_{D_{TR}|D_{CR}}$, with respect to *D*_*CR*_, are equal to the marginal means Δ_*TC*_ and Δ_*TR*_, assuming *D*_*CR*_ follows a normal distribution with mean Δ_*CR*_ and variance $\sigma_{D_{CR}}^{2}$. While the difference (*D*_*CR*_ − Δ_*CR*_) would be reduced by increasing the size of the TCR trial, such as the number of sites, the background effect *D*_*CR*_ in expectation (i.e. Δ_*CR*_) can never be reduced because the same control appears in all sites. Our objective is to find a measure of the trait effect less-dependent, or ideally independent, of the background effect.

Although $\mu_{D_{TR}|D_{CR}}$ is a regression-type of estimate, *D*_*CR*_ is a random variable which can take different values, making it different from classical regression. Let *d*_*TC*_, *d*_*TR*_, and *d*_*CR*_ be values of *D*_*TC*_, *D*_*TR*_, and *D*_*CR*_ estimated from a TCR trial. Two cases of special interest in (3) and (4) are when *D*_*CR*_ = *d*_*CR*_ and *D*_*CR*_ = 0, where *D*_*CR*_ = *d*_*CR*_ assumes the control background as in the current trial, and *D*_*CR*_ = 0 implies that the random control background is adjusted to the mean of the reference distribution.

$$\left\{ \begin{array}{l} \mu_{D_{TR}|D_{CR}=d_{CR}}=\mu_{D_{TC}|D_{CR}=d_{CR}}+d_{CR}=\Delta_{TC}+d_{CR}-\frac{\sigma_{D_{TC}}^{2}}{{2\sigma }_{D_{CR}}^{2}}(d_{CR}-\Delta_{CR})\\ \mu_{D_{TR}|D_{CR}=0}=\mu_{D_{TC}|D_{CR}=0}=\Delta_{TC}+\frac{\sigma_{D_{TC}}^{2}}{{2\sigma }_{D_{CR}}^{2}}\Delta_{CR} \end{array}\right.$$ (5)

Intuitively, (5) is a regression of *D*_*TR*_ on *D*_*CR*_. $\sigma_{D_{TC}}^{2}/2\sigma_{D_{CR}}^{2}$ is the regression coefficient based on the correlation structure, $\mu_{D_{TR}|D_{CR}=0}$ represents the expectation of the sample estimate of the intercept (or the expected mean of *D*_*TR*_ at *D*_*CR*_ = 0), and $\mu_{D_{TR}|D_{CR}=d_{CR}}$ represents the expectation of *D*_*TR*_ given *D*_*CR*_ at the observed mean *d*_*CR*_ in the current study. The implication of $\mu_{D_{TR}|D_{CR}=d_{CR}}$ will be discussed later in (8) and (9). The intercept $\mu_{D_{TR}|D_{CR}=0}$ is more interesting here; it has two components: the first part is Δ_*TC*_, known to be a pure estimate of the GM trait effect; and the second part is a portion of Δ_*CR*_, due to the covariance structure defined in property [b]. This is the familiar form of a simple regression: the intercept, say *α*, is equal to (*μ*_*y*_ − *βμ*_*x*_) [10]; i.e. the dependent variable mean (*μ*_*y*_) minus the regression coefficient (*β*) times the independent variable mean (*μ*_*x*_). Here, the correlation and the regression coefficient are negative. The second part of (5) separates $\mu_{D_{TR}|D_{CR}=0}$ from Δ_*TC*_ as a potentially better measure of the trait effect. It’s shown later when and how much $\mu_{D_{TR}|D_{CR}=0}$ would make a maximum difference as compared with Δ_*TC*_ and Δ_*TR*_.

The implication of (5) is technically-interesting and practically-important. By conditioning, three parameters of (Δ_*TC*_, Δ_*TR*_, Δ_*CR*_) are resolved into a two-dimensional space of ($\mu_{D_{TR}|D_{CR}=0}$, Δ_*CR*_). In this two-dimensional space, the value of $\mu_{D_{TR}|D_{CR}=0}$ is determined mostly by Δ_*TC*_ and depends on Δ_*CR*_ only to the extent of the correlation between *D*_*TC*_ and *D*_*CR*_. Besides, given the background, the expectations of *D*_*TC*_ and *D*_*TR*_ are the same. Thus, from a statistical point of view, $\mu_{D_{TR}|D_{CR}=0}$ or $\mu_{D_{TC}|D_{CR}=0}$ can be considered as the unique parameter of interest to replace Δ_*TC*_ and Δ_*TR*_ in the comparative assessment.

### A trait model for the TCR trial

Characteristics of the TCR trial have posed some difficulties [3, 4] in statistical modelling, partly due to properties [a] and [b], and because of the differential field arrangement of the test and the control materials, versus references, among sites. At least three linear mixed models have been proposed for the TCR trial. The discussion of these models is delayed until later, but two common limitations are noteworthy (which were also concerns of the agricultural biotechnology industry [4]). The first limitation is the inability to decompose the trait effect and random background effect; these effects are therefore confounded in the equivalence testing. In fact, EFSA requires a two-step procedure—using EFSA Model 1 (assuming both the trait difference and the control background effect as fixed) for difference testing and Model 2 (assuming the independent random background effect separately for the test and the control) for setting equivalence limits [2]. The second limitation is the inability to separate the GM trait-related interaction from the control- and reference-related ones, which are all inherent to the design of the TCR trail. A GM trait-related interaction of two nearly-isogenic lines (i.e. the test and the control materials) with the site, if present, would certainly be different from a G×E interaction in a conventional plant breeding context. Even though the interaction may not always be an important source of variation, the statistical model should provide such an option, if needed. Instead, EFSA requests a separate, *post hoc* interaction analysis, restricting data to test and control only, and assuming the site effect is fixed [2]. These limitations are structural shortcomings of the existing methods. For this reason, a trait model is proposed, which defines effects on linear contrasts of genotype groups corresponding to either the trait- or control background effect. This contrasts with the current genotype models which define effects on each genotype group.

Two factors can be defined on the three genotype groups based on property [a]: the GM trait *T* with two levels (GM: the test material, and Non-GM: the control material and the references), and the background *G* also with two levels (Non-Ref: The test and the control varieties, and Ref: reference varieties). Then, *G* and *T*(*G*) represent two orthogonal, single-degree-of-freedom decompositions of the genotype group effects. While *G* represents the contrast of the test and the control as a pair versus the references, *T*(*G*) is the contrast of the test versus the control within the Non-Ref group.

The trait model can be expressed as:

Y=μ+S+B(S)+G+T(G)+GS+TS(G)+g+e Model A

where *Y* is the observed response; *μ* is overall mean; [*S* and *B*(*S*)] are environmental effects for site and block within each site, *g* is the background effect, and *e* is the residual. *G* and *T*(*G*) are main effects, and *GS* and *TS*(*G*) are corresponding interactions. Based on property [a] of the TCR trial, the background effect is defined as:

$$g=\left\{ \begin{array}{l} Control\;material,\;if\;g\;is\;in\;the\;test\;\mathrm{or}\;\mathrm{the}\;\mathrm{control}\;\mathrm{group}\\ Individual\;reference\;variety,\;if\;g\;is\;from\;reference\;group \end{array}\right.$$

Only *μ*, *G* and *T*(*G*) are assumed to be fixed; all other factors are random and assumed to be independently distributed with mean 0 and corresponding variance. One advantage of the expression of Model A over the model equations (one equation for each genotype) in Kang and Vahl [5] and Vahl and Kang [8] is the avoidance of a specified covariance structure derived from the random common background effect between the test and the control materials.

The primary factor differentiating Model A from the existing methods can be summarized as follows. First, Model A assumes, in the effect *g*, that the random control background is identical for the control and the test, and has the same variance as the references, which is part of property [a] of the TCR trial. Second, there are three possible types of interactions in the TCR trial: the GM trait-by-site *TS*(*G*), the control background-by-site *GS*, and the reference-by-site. Inclusion or exclusion of each or all of them is optional depending on the data. Model A includes the first two types of interactions because of the balanced arrangement of the test and control materials across sites, which support the inclusion of the trait-by-site and the control background-by-site interactions in the analysis. A minor modification could also include the reference-by-site interaction, when a proper field arrangement of references is available across sites. Since our focus here is on modelling and estimating effects *G* and (*G*), and interactions *GS* and *TS*(*G*), Model A is sufficient. Additional discussion is provided in subsequent sections.

The components of variances and co-variances are as follows in a design with the same number, but different varieties of references, at each site. Overlapping references among sites will be discussed later. Let *n*_*s*_ denote the number of sites, *n*_*b*_ the number of blocks and *n*_*sg*_ the number of references at each site, and *n*_*g*_ the total number of references.

$$\left(\begin{array}{c} \sigma_{D_{TC}}^{2}\\ \sigma_{D_{CR}}^{2}\\ \sigma_{D_{TR}}^{2}\\ \sigma_{D_{TC},D_{CR}}\\ \sigma_{D_{TC},D_{TR}}\\ \sigma_{D_{CR},D_{TR}} \end{array}\right)=\left(\begin{array}{c} {2\sigma }_{TS(G)}^{2}/n_{s}+2\sigma_{e}^{2}/\left(n_{b}n_{s}\right)\\ {{2\sigma }_{GS}^{2}/n_{s}+2\sigma }_{TS(G)}^{2}/n_{s}+\sigma_{g}^{2}+\sigma_{g}^{2}/n_{g}+\sigma_{e}^{2}/\left(n_{b}n_{s}\right)+\sigma_{e}^{2}/\left(n_{b}n_{sg}n_{s}\right)\\ {{2\sigma }_{GS}^{2}/n_{s}+2\sigma }_{TS(G)}^{2}/n_{s}+\sigma_{g}^{2}+\sigma_{g}^{2}/n_{g}+\sigma_{e}^{2}/\left(n_{b}n_{s}\right)+\sigma_{e}^{2}/\left(n_{b}n_{sg}n_{s}\right)\\ -\sigma_{TS(G)}^{2}/n_{s}-\sigma_{e}^{2}/\left(n_{b}n_{s}\right)\\ \sigma_{TS(G)}^{2}/n_{s}+\sigma_{e}^{2}/\left(n_{b}n_{s}\right)\\ {2\sigma }_{GS}^{2}/n_{s}+\sigma_{TS(G)}^{2}/n_{s}+\sigma_{g}^{2}+\sigma_{g}^{2}/n_{g}+\sigma_{e}^{2}/\left(n_{b}n_{sg}n_{s}\right) \end{array}\right)$$ (6)

where $\sigma_{GS}^{2},\;\sigma_{TS(G)}^{2},\;\sigma_{g}^{2}$, and $\sigma_{e}^{2}$ are corresponding variance components of *GS* and *TS*(*G*), *g* and *e*, respectively, in Model A. A simplified case may help the understanding of (6) when no G×E is assumed, i.e. $\sigma_{GS}^{2}=0$ and $\sigma_{TS(G)}^{2}=0$. Then, (6) becomes

$$\left(\begin{array}{c} \sigma_{D_{TC}}^{2}\\ \sigma_{D_{CR}}^{2}\\ \sigma_{D_{TR}}^{2}\\ \sigma_{D_{TC},D_{CR}}\\ \sigma_{D_{TC},D_{TR}}\\ \sigma_{D_{CR},D_{TR}} \end{array}\right)=\left(\begin{array}{c} 2\sigma_{e}^{2}/\left(n_{b}n_{s}\right)\\ {(1+1/n_{g})\sigma }_{g}^{2}+\sigma_{e}^{2}/\left(n_{b}n_{s}\right)+\sigma_{e}^{2}/\left(n_{b}n_{sg}n_{s}\right)\\ (1+1/n_{g})\sigma_{g}^{2}+\sigma_{e}^{2}/\left(n_{b}n_{s}\right)+\sigma_{e}^{2}/\left(n_{b}n_{sg}n_{s}\right)\\ {-\sigma }_{e}^{2}/\left(n_{b}n_{s}\right)\\ \sigma_{e}^{2}/\left(n_{b}n_{s}\right)\\ (1+1/n_{g})\sigma_{g}^{2}+\sigma_{e}^{2}/\left(n_{b}n_{sg}n_{s}\right) \end{array}\right)$$ (7)

Results in (7) show that under Model A, $\sigma_{D_{TC}}^{2}$ is the same as that of EFSA Model 1, but $\sigma_{D_{TR}}^{2}$ under Model A is the same as that of EFSA Model 2. This is because Model A includes not only the fixed trait effect but also the random background of the test and the control, i.e. the test and the control materials are assumed to be both fixed and random per the effect, rather than the identity of the genotype group.

When fitting Model A, (*d*_*TC*_, *d*_*CR*_, *d*_*TR*_) are the sample means. Let $s_{d_{TC}}^{2}$ and $s_{d_{CR}}^{2}$ and $s_{d_{TC},d_{CR}}$ be the sample variances and covariance. When these estimates are applied to Eq in (5), the sample estimates of the conditional means can now be obtained:

$$\left\{ \begin{array}{l} {\hat{\mu }}_{D_{TR}|D_{CR}=d_{CR}}=d_{TR}=d_{TC}+d_{CR}\\ {\hat{\mu }}_{D_{TR}|D_{CR}=0}=d_{TC}+d_{CR}{∙s}_{d_{TC}}^{2}/\left(2s_{d_{CR}}^{2}\right) \end{array}\right.$$ (8)

Eq (8) indicates that *d*_*TR*_ is the estimate of Δ_*TR*_ given *D*_*CR*_ at its observed value *d*_*CR*_. It simply says that, given the control background as *d*_*CR*_, the observed trait difference *d*_*TR*_ would be a sum of two correlated differences *d*_*TC*_ and *d*_*CR*_, which is what EFSA uses in their equivalence testing [2]. Given *D*_*CR*_ = 0, however, ${\hat{\mu }}_{D_{TR}|D_{CR}=0}$ is only slightly affected by *d*_*CR*_. It can be shown that the correlation between ${\hat{\mu }}_{D_{TR}|D_{CR}=0}$ (the estimate of the so-defined GM trait effect $\mu_{D_{TR}|D_{CR}=0}$) and *d*_*CR*_ (the estimate of the background effect Δ_*CR*_) is zero. That is, they are independent from each other when normality is assumed. Now, we have two independent estimates for two parameters, one for the background effect and another for the GM trait effect.

To estimate the variance of ${\hat{\mu }}_{D_{TR}|D_{CR}=0}$, the variance and co-variance of *d*_*TC*_ and *d*_*CR*_ can be obtained directly from (6) or (7). Even though the ratio $s_{d_{TC}}^{2}/{2s}_{d_{CR}}^{2}$ is a random variable itself, its variance consists of higher order terms and the variances of *d*_*TC*_ and *d*_*CR*_ are the leading terms in the overall variance of ${\hat{\mu }}_{D_{TR}|D_{CR}=0}$. Therefore, an approximation can be obtained based on the variation of *d*_*TC*_ and *d*_*CR*_:

$$\left\{ \begin{array}{l} s_{{\hat{\mu }}_{D_{TR}|D_{CR}=d_{CR}}}^{2}=s_{d_{TR}}^{2}\\ s_{{\hat{\mu }}_{D_{TR}|D_{CR}=0}}^{2}=s_{D_{TR}|D_{CR}}^{2}=s_{d_{TC}}^{2}\left(1-\frac{s_{d_{TC}}^{2}}{4s_{d_{CR}}^{2}}\right) \end{array}\right.$$ (9)

Note that $s_{{\hat{\mu }}_{D_{TR}|D_{CR}=0}}^{2}$ has the same form as $\sigma_{D_{TR}|D_{CR}}^{2}$ in (4) with each variance replaced by the corresponding sample estimates due to the nature of the regression type of estimation. Thus, $s_{D_{TR}|D_{CR}}^{2}$ denotes the sample estimate of the variance of the conditional difference. Though *d*_*CR*_ is the control background effect of a TCR trial and could never exactly equal zero, by property [a], *d*_*CR*_ is random and follows a distribution with mean zero and variance $\sigma_{D_{CR}}^{2}$ estimated from the references.

Since both *D*_*TR*_ and *D*_*CR*_ are the respective differences from the same reference mean, the regression of *D*_*TR*_ on *D*_*CR*_ is then equivalent to the regression of the test mean on the control mean. The conditional mean ${\hat{\mu }}_{D_{TR}|D_{CR}=0}$ is the test mean given the control mean at the estimated reference mean. Therefore, ${\hat{\mu }}_{D_{TR}|D_{CR}=0}$ would have minimum variance among estimates for given *D*_*CR*_ at any value other than zero. Furthermore, the sample variance $s_{{\hat{\mu }}_{D_{TR}|D_{CR}=0}}^{2}$ would have the same form as the conditional variance of (3) and (4), i.e. $s_{{\hat{\mu }}_{D_{TR}|D_{CR}=0}}^{2}=s_{D_{TR}|D_{CR}}^{2}$. Both (8) and (9) are likelihood estimates of the corresponding parameter functions since each component is the likelihood estimate from the mixed model analysis.

Now, the following hypothesis can be tested using a t-test:

$$H_{0}:\;\mu_{D_{TR}|D_{CR}=0}=0\;vs.\;H_{1}:\mu_{D_{TR}|D_{CR}=0}\neq 0$$ (10)

$$t_{D_{TR}|D_{CR}}=\frac{{\hat{\mu }}_{D_{TR}|D_{CR}=0}}{s_{{\hat{\mu }}_{D_{TR}|D_{CR}=0}}}=\frac{d_{TC}+d_{CR}{∙s}_{d_{TC}}^{2}/\left(2s_{d_{CR}}^{2}\right)}{\sqrt{s_{d_{TC}}^{2}(1-s_{d_{TC}}^{2}/4s_{d_{CR}}^{2})}}$$ (11)

with degrees of freedom (${df}_{d_{TC}}-1)$. When the Kenward-Rodger [10, 11] method is applied, the degrees of freedom can be obtained from the analysis for ${df}_{d_{TC}}$, and an additional adjustment of minus one is due to the regression.

## Results

In this section, the asymptotic and sample performance of the conditional method is evaluated for a wide range of parameter values. Simulations are conducted on selected parameter sets to compare the conditional method and trait model with the EFSA method. Lastly, the EFSA example [2] on maize grain composition data is reanalyzed using the conditional method and results are compared. For the numerical evaluation and simulation, a typical TCR trial was assumed, i.e. *n*_*s*_ = 8, *n*_*b*_ = 4, *n*_*gs*_ = 4. For illustration of the conditional method, all references were assumed different, i.e. *n*_*g*_ = *n*_*s*_*n*_*gs*_. The variation setting was defined as the ratio between the reference (or genetic) versus residual variances $\sigma_{g}^{2}:\sigma_{e}^{2}$. Zero G×E interactions were assumed in data generation for simulations.

### Asymptotic and sample performance of the conditional method

First, the conditional and unconditional variances are compared, and the correlations are examined as functions of the variation settings. From Eqs (4) and (8), the asymptotic correlation coefficients can be calculated as:

$$\left\{ \begin{array}{l} \rho_{D_{TC},D_{TR}|D_{CR}=0}=\sqrt{1-\sigma_{D_{TC}}^{2}/2\sigma_{D_{CR}}^{2}}\\ \rho_{D_{TR},D_{TR}|D_{CR}=0}=\frac{\sigma_{D_{TC}}}{\sigma_{D_{CR}}}\sqrt{1-\sigma_{D_{TC}}^{2}/2\sigma_{D_{CR}}^{2}} \end{array}\right.$$ (12)

**Fig 2:**
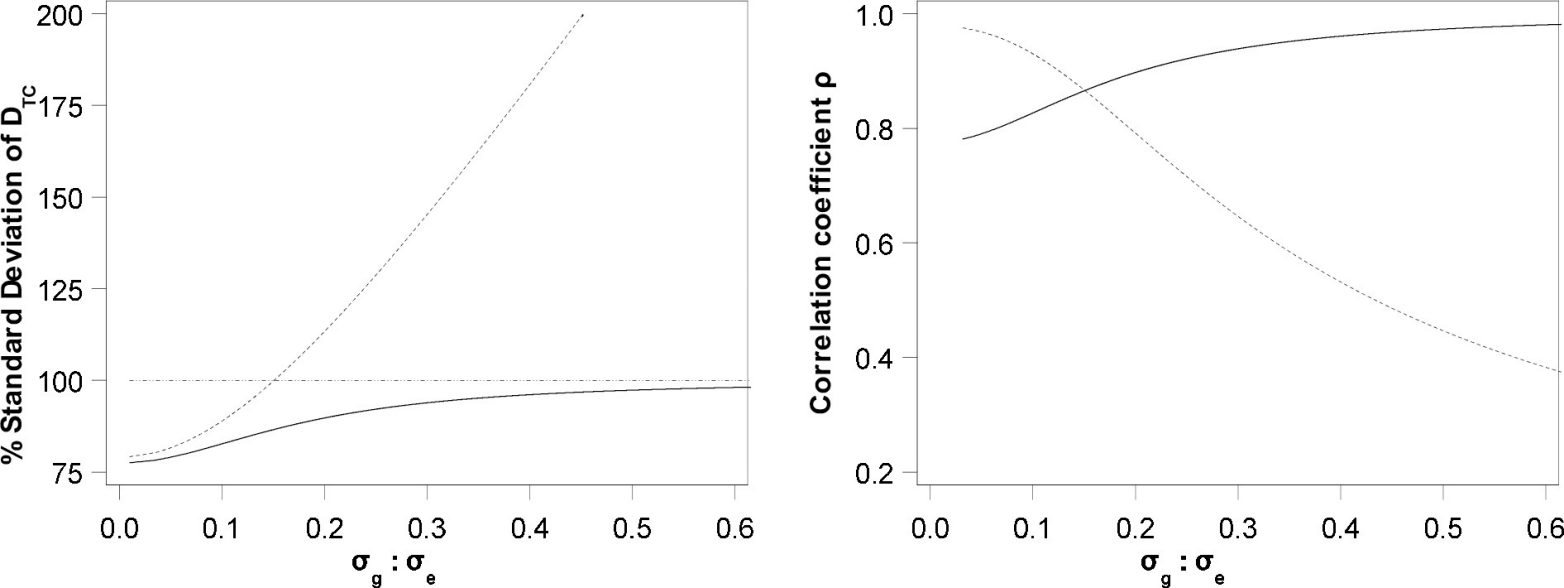
Standard deviations of simple and conditional trait differences and their correlation. Standard deviations (Left) of $\sigma_{D_{TR}}$ (dash) and $\sigma_{D_{TR}|D_{CR}=0}$ (solid) relative to $\sigma_{D_{TC}}$ (100% dot line), and correlation coefficients (Right) of $\rho_{D_{TC},D_{TR}|D_{CR}=0}$ (dash) and $\rho_{D_{TR},D_{TR}|D_{CR}=0}$ (solid), as functions of *σ*_*g*_: *σ*_*e*_.

Fig 2 illustrates the relationship of standard deviations $\sigma_{D_{TR}}$ and $\sigma_{D_{TR}|D_{CR}=0}$ relative to $\sigma_{D_{TC}}$, and of correlation coefficients $\rho_{D_{TC},D_{TR}|D_{CR}=0}$ and $\rho_{D_{TR},D_{TR}|D_{CR}=0}$, as functions of *σ*_*g*_: *σ*_*e*_. A few results are worth noting. In general, $\mu_{D_{TR}|D_{CR}=0}$ has the lowest variance as compared with *D*_*TC*_ and *D*_*TR*_. Even though the variation of *D*_*TR*_ is mostly larger than that of *D*_*TC*_ due to the background effect, it is actually smaller when *σ*_*g*_ is small. When genotypic variation is negligible, the residual becomes the main source of variability even among references. As the total number of replicates of references is larger than that of the control (a common case in practice), $\sigma_{D_{TR}}^{2}<\sigma_{D_{TC}}^{2}$ becomes true, which is also revealed by Eqs (6) and (7).

**Fig 3:**
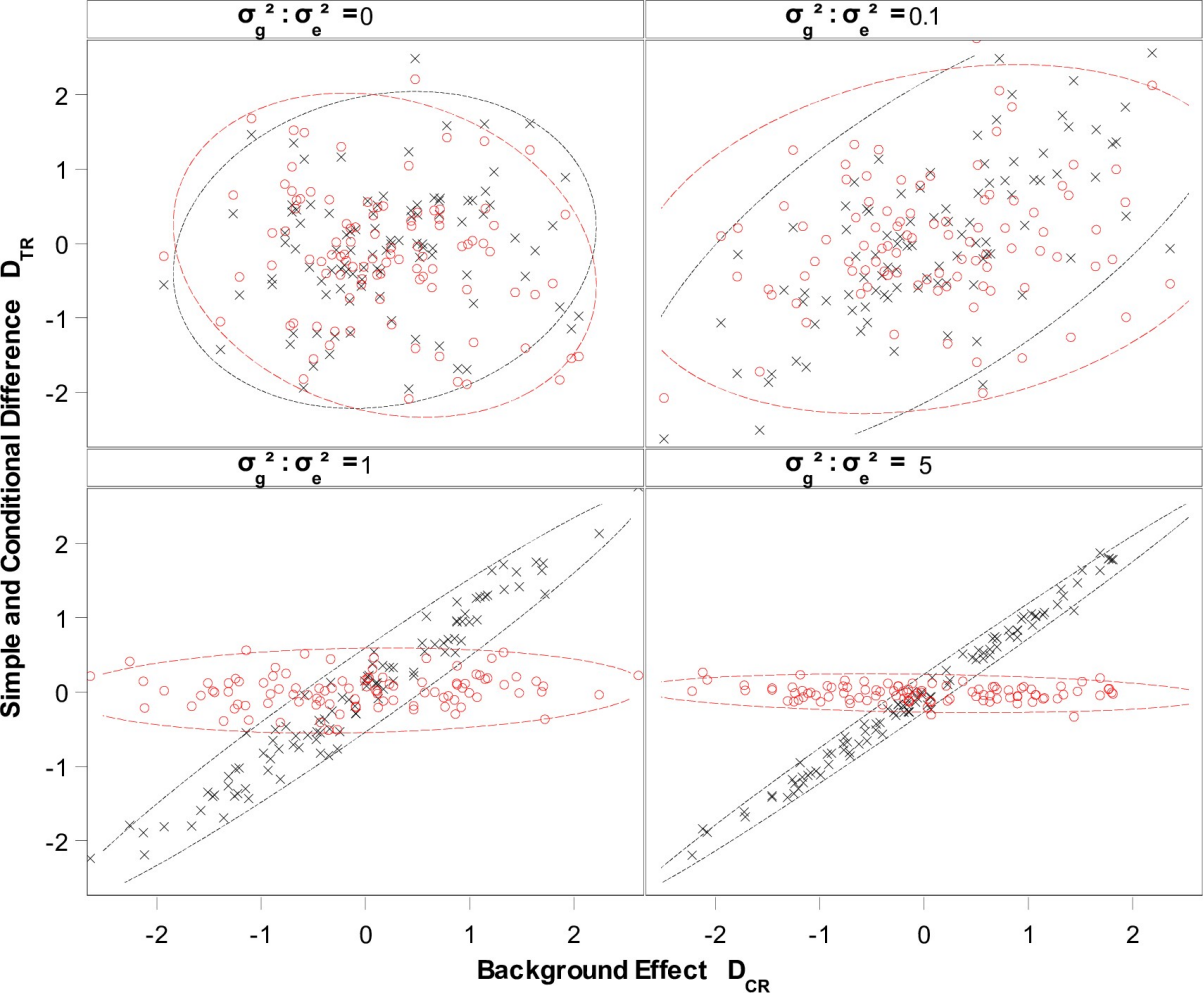
Scatter plots of simple and conditional trait differences as functions of the background effect. 100 simulated mean differences *D*_*TR*_ (in Black) and conditional mean difference $\mu_{D_{TR}|D_{CR}=0}$ (in Red) versus the background effect *D*_*CR*_ in the unit of $\sigma_{D_{CR}}$ and 95% prediction ellipses for each setting of $\sigma_{g}^{2}:\sigma_{e}^{2}=(0,\;0.1,\;1,\;5)$.

Sample performance of the conditional mean is examined in Fig 3. Fig 3 shows 100 pairs of simulated values of *D*_*TR*_ and ${\hat{\mu }}_{D_{TR}|D_{CR}=0}$ versus *D*_*CR*_, all scaled by $\sigma_{D_{CR}}$ (or $\sigma_{D_{TR}}$ equivalently) to provide an intuitive impression of the effect of conditioning. Four variation settings $\sigma_{g}^{2}:\sigma_{e}^{2}=(0,\;0.1,\;1,\;5)$ were assumed by fixing $\sigma_{e}^{2}=1$ and adjusting the value of $\sigma_{g}^{2}$ accordingly. The Least-Square Mean difference, *D*_*TR*_, shows positive parallel change with *D*_*CR*_ due to the common background, as long as a $\sigma_{g}^{2}$ is large enough, say $\sigma_{g}^{2}\geq 0.1\;\sigma_{e}^{2}$, or $\sigma_{g}^{2}:\sigma_{e}^{2}\geq 0.1$. The parallel change becomes dramatic as $\sigma_{g}^{2}:\sigma_{e}^{2}$ increases to 1. The conditional mean ${\hat{\mu }}_{D_{TR}|D_{CR}=0}$, however, shows no correlation with the background. Results in Fig 3 strongly support the message in Fig 1 that EFSA, by using *D*_*TR*_ as a test statistic, is mostly testing the background effect, as long as nonnegligible reference variation exists. In contrast, the conditional method using ${\hat{\mu }}_{D_{TR}|D_{CR}=0}$ is independent of the background, and is thus a true test of the trait effect even if the reference variation is around zero.

### Conditional difference testing

In this section, the practical performance of the difference testing methods is compared using the following hypotheses:

$$\left\{ \begin{array}{l} H_{01}:\;\Delta_{TC}=0\;vs.\;H_{11}:\Delta_{TC}\neq 0\\ H_{02}:\;\Delta_{TR}=0\;vs.\;H_{12}:\Delta_{TR}\neq 0\\ H_{03}:\;\mu_{D_{TR}|D_{CR}=0}=0\;vs.\;H_{13}:\mu_{D_{TR}|D_{CR}=0}\neq 0 \end{array}\right.$$ (13)

Although hypotheses (13) are for difference testing, the results are directly related to equivalence testing, as is shown next.

The empirical Type I error of the difference testing was evaluated by simulation at *α* = 0.1 (the same as in [2]). Unlike those results in Fig 2, simulations are subject to sampling variation. The total variation mirrored the example in [2] and assumed a coefficient of variation (CV) of 40%, including both the genetic and residual components with an overall mean 10 and variation setting $\sigma_{g}^{2}:\sigma_{e}^{2}$ the same as in Fig 3. The site variation was assumed to be $\sigma_{e}^{2}/4$ with no block variation. For the power analysis, the trait effect was varied from 0 to 3, which is equal to 0 to 0.75 standard deviations of the total effects of the reference and residual. The trait model and the conditional method were applied to each simulated dataset and the Kenward-Roger adjustment was applied in the difference testing. Simulations were repeated 10,000 times for each set of parameter values, and results were summarized over replicate simulations.

**Fig 4:**
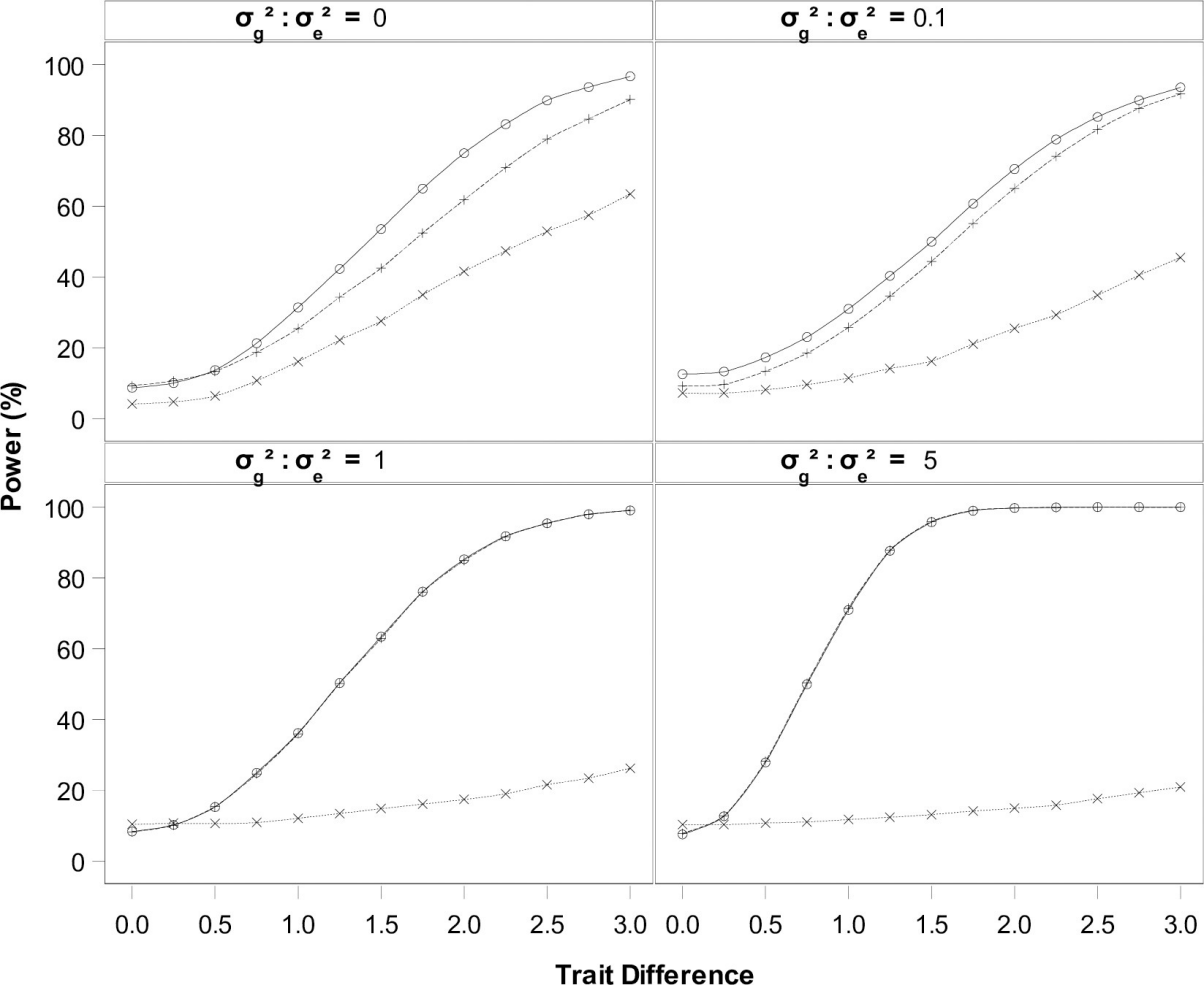
Empirical power of three difference tests. Statistical power of testing *H*_01_: Δ_*TC*_ = 0 (dash), *H*_02_: Δ_*TR*_ = 0 (dot), $H_{03}:\;\mu_{D_{TR}|D_{CR}=0}=0$ (solid) under four variation settings. Markers represent the simulation results, and the curves were generated as smooth lines by SAS procedure SGPLOT [12].

All three methods performed as expected with the Type I error being around the nominal level (approximately 7 to 13% except one case *H*_02_: Δ_*TR*_ = 0 and $\sigma_{g}^{2}:\sigma_{e}^{2}=0$) (Table 1). Slight deviations from the nominal level are likely due to the bias in the likelihood estimates of the variance components; especially when the actual value is close to zero, the non-zero restriction is applied in the estimation, the assumed experimental design will dictate the degrees of freedom of the variance estimates, as well as potential biases. Results of the power analysis in Fig 4 confirm those from Figs 1–3: among the three difference tests, the conditional difference test $H_{03}:\;\mu_{D_{TR}|D_{CR}=0}=0$ is always the most efficient, and the simple difference test *H*_02_: Δ_*TR*_ = 0 is often the least efficient. When $\sigma_{g}^{2}:\sigma_{e}^{2}$ is low, say < 0.1, the difference in statistical power is substantial between testing for *H*_01_: Δ_*TC*_ = 0 and $H_{03}:\;\mu_{D_{TR}|D_{CR}=0}=0$. As $\sigma_{g}^{2}:\sigma_{e}^{2}$ gets larger, such as $\sigma_{g}^{2}:\sigma_{e}^{2}>1$, this difference diminishes, and the two tests become very similar.

**Table 1:** The empirical Type I errors of difference testing under the trait model assuming Δ_*TC*_ = 0 at *α* = 0.10.

$\sigma_{g}^{2}:\sigma_{e}^{2}$	Type I Error for testing *H*_0_:		
	Δ_*TC*_ = 0	Δ_*TR*_ = 0	$\mu_{D_{TR}|D_{CR}=0}=0$
0	9.43	3.92	8.61
0.1	9.60	7.02	12.74
1	8.43	10.10	8.51
5	7.79	10.32	7.5

Results in Fig 4 are directly relevant to those of EFSA’s equivalence testing. Large background variation in *D*_*TR*_ often makes the testing *H*_02_: Δ_*TR*_ = 0 very inefficient with extremely low power, regardless of the trait difference. This implies that, when the trait difference is small, large variation in *D*_*TR*_ will result in a failure to reject the non-equivalence hypothesis at a rate much greater than the nominal α = 0.10 level. Alternatively, a large trait effect might confound with the background variation, resulting in a rejection of the non-equivalence hypothesis.

### Revisiting EFSA

The two-step difference and equivalence testing approach was illustrated with a maize grain composition example [2, 3]. The purpose of revisiting that example here is to explore the performance of the trait model and conditional method. Before the re-analysis, results reported in EFSA (2010) were confirmed, including the *post hoc* testing of the trait-by-site interaction.

In the re-analysis with the trait model, SAS PROC GLIMMIX [12] was used since the procedure provides a likelihood ratio test on variance components using option COVTEST. One statistically-significant interaction (Neutral Detergent Fiber) was found at the 0.05 level (the same as used by EFSA) for the trait-by-site interaction, and none of the control background-by-site interactions were significant. In contrast, eight statistically-significant interactions were found using the EFSA method [2]—treating site as a fixed effect while restricting the data to test and control only (i.e., references are excluded). The discrepancy highlighted here may be the result of the data or the statistical procedure. Nevertheless, the interaction is an effect of the design, and should be reflected in the statistical model (as in the trait model) instead of in a fragmented, *post hoc* procedure, as recommended by EFSA [2].

EFSA could not reach a conclusion on equivalence for nine of the 53 analytes (including two analytes with reference variance estimated as zero) [2]. Among those analytes, only two were found to be significantly-different from the control (Table 2). Using *s*_*g*_ estimated from the corresponding model, the standardized differences *d*_*TR*_/*s*_*g*_ of the seven analytes under the EFSA model are all greater than or close to 2.0 (from 1.92 to 2.75) in absolute value. Under the trait model, five of the seven show a background effect *d*_*CR*_/*s*_*g*_ greater than 2 in absolute value. Obviously, the inability to reach conclusions on equivalence is the result of large control background effects, and has nothing to do with the GM trait itself. In the conditional difference testing, nine analytes showed a statistically-significant difference at the α = 0.10 level. The standardized differences ${\hat{\mu }}_{D_{TR}|D_{CR}=0}/s_{g}$ are mostly less than 1 in absolute value and only two (Niacin and Vitamin B2) were slightly greater than 1 (ranging from 0.27 to 1.38 except Phytic Acid with reference variation estimated as zero).

**Table 2:** Reanalysis of EFSA example by the conditional method and comparing results with those of EFSA method.

Analyte	Estimates of EFSA method		Estimates of conditional method		
	*d*_*TC*_/*s*_*g*_	*d*_*TR*_/*s*_*g*_	$s_{g}^{2}:s_{e}^{2}$	*d*_*CR*_/*s*_*g*_	${\hat{\mu }}_{D_{TR}|D_{CR}=0}/s_{g}$
16:0 Palmitic	0.26	2.57*	4.12	2.31	0.31
18:1 Oleic	0.39*	0.78	9.58	0.38	0.40*
18:3 Linolenic	-0.87*	-0.69	3.11	0.18	-0.84*
22:0 Behenic	-0.54*	-0.23	4.31	0.30	-0.52*
Lysine	1.57*	-2.75*	0.33	-4.44	0.85
Magnesium	0.49*	-0.44	3.58	-0.93	0.47*
Manganese	0.27*	0.03	9.23	-0.24	0.27*
Niacin	-1.22*	-2.44*	5.03	-1.22	-1.24*
Phosphorus	0.02	-1.92*	2.00	-2.03	-0.05
Potassium	-0.40	-2.29*	1.95	-1.97	-0.52
Proline	0.46*	-0.33	5.45	-0.80	0.45*
Vitamin B2	-1.24*	-1.96	0.35	-0.71	-1.38*
Vitamin B6	-0.03	-2.05*	3.35	-2.02	-0.08
Vitamin E	-0.30	-2.29*	2.69	-2.03	-0.36

Analytes included are those showing significance in EFSA difference testing and failure of concluding equivalence (nine in total including two excluded due to zero reference variation) in the equivalence testing, and significance in the conditional difference (nine in total with Phytic Acid excluded due to zero reference variance in both methods) testing at the *α* = 0.10 level. The estimated differences were standardized by the reference standard deviation estimated by respective model. Asterisks ‘*’ denote significant differences and lack of equivalences.

## Discussion

The equivalence testing method prescribed by [2] assesses differences between the test and references. These differences, as the main topic of this paper, may be driven by a trait effect, a control background effect, or both. The mixed models proposed in the literature and adopted by EFSA confound the trait and control background effects. Two direct consequences are worth noting. The first is the mismatched outcome type (i.e. non-significance in the difference testing and non-equivalent to the conventional references) that may result in the two-step procedure—using Δ_*TC*_ in difference testing and Δ_*TR*_ in equivalence testing. Second, if the control background effect is large, the result of EFSA equivalence testing has little or nothing to do with the trait effect, which should be the sole focus of the comparative assessment. The proportion of background variation in the test-reference difference can be estimated as the variation reduction from the conditioning:

$$R_{D_{TR}|D_{CR}}^{2}=1-\frac{\sigma_{D_{TR}|D_{CR}}^{2}}{\sigma_{d_{TR}}^{2}}\;\approx {\left(1-\frac{\sigma_{d_{TC}}^{2}}{2\sigma_{d_{CR}}^{2}}\right)}^{2}$$

For example, when $\sigma_{g}^{2}:\sigma_{e}^{2}=1,\;R_{D_{TR}|D_{CR}}^{2}$ will be as large as 94%. Since the background effect is often random among different endpoints, so is the testing result. As the re-analysis of the EFSA example indicates, $\sigma_{g}^{2}:\sigma_{e}^{2}$ has a mean estimate of 3.4 and a range of 0 to 9.6, and the mean of $R_{D_{TR}|D_{CR}}^{2}$ is 86%. In addition, seven out of nine analytes, which could not be concluded as either equivalent or non-equivalent, have relatively large control background effects, while the trait differences are small. Experimental results have recently been provided directly relevant to this observation [13]. They plotted means of GM stacks (i.e. materials with more than one GM trait) and references from a group of studies against the corresponding control mean in each study with different background and concluded that traditional breeding more strongly influenced grain composition than did transgenesis or crossing (stacking) GM events. Therefore, confounding the GM trait effect with natural genotypic variation is not appropriate for comparative assessment and is a misinterpretation of the Codex principle of substantial equivalence. In contrast, the conditional method proposed here evaluates the conditional mean difference, assuming a background effect of zero; this is equivalent to statistically-centering the control background at the reference mean. Consequently, the comparative assessment is independent of the natural genomic differences between the control and references and is more efficient than the EFSA method in the event of a real trait effect.

### G×E effects in the TCR trial

The lack of a genotype-by-environment interaction term is one of the main concerns highlighted by Ward et al. [4]. In fact, three types of genotype-by-environment interactions (G×E) can be defined based on the design of the generic TCR trial: the GM trait-by-site, the control background-by-site, and the references-by-site. The trait model proposed in this investigation can accommodate all or any subset of the interactions based on evidence in the data. One consequence among various models is that the trait-by-site interaction is the leading term in the standard error of the estimated trait difference under the trait model. The presence or absence of this interaction term often affects the error degrees of freedom used in testing the trait difference, even if the true trait effect is negligible or absent. This is likely why only eight significant trait differences were detected under the trait model, while 22 analytes were significantly-different under EFSA Model (1) [2]. As illustrated in [4], by neglecting to include any interaction terms, the difference tests conducted under EFSA Model (1) will always be based on an error term that mixes all potential sources of interaction and the residual with a correspondingly large degrees of freedom. If the GM trait-by-site and reference-by-site interactions are both non-zero and of similar magnitude, the error term is under-estimated and the associated degrees of freedom tends to be higher (relative to the true distribution of the test statistic), leading to an inflated false positive error rate that exceeds the nominal level. Other circumstances may result in the difference test being overly conservative. In fact, the only time when the difference test performs as intended under [2] is when all interactions involving site are zero [4].

The control background-by-site interaction may not be of interest to the GM safety assessment *per se*. It is, however, a component in the covariance structure, and will affect the standard error of the estimated background effect. The impact of this interaction must be determined on a case-by-case basis.

The reference-by-site interaction is different from the above two types of interactions. First, the TCR design does not require overlapping references among sites. Without overlapping references, this interaction cannot be properly estimated, and a good estimate of the interaction depends on a large degree of overlap. Second, the reference-related interaction will confound with the reference variation when absent in the statistical model. Such confounding should not have any consequence on the comparative assessment.

### Equivalence criterion

The equivalence of the test and references should be determined in the context of natural variation, i.e. reference variation, when reference variation is a significant part of the total variation. When the reference variation is estimated as zero, EFSA (declares the equivalence criterion and the equivalence status as undetermined [2]. Presumably, when it is near zero relative to the residual variation, the equivalence criterion and the conclusion would be poorly determined. In practice, many endpoints have negligible reference variance. For example, the ratio of $\sigma_{g}^{2}:\sigma_{e}^{2}$ was estimated between 0 to 9.6 across 53 analytes in the EFSA example under Model A. It makes sense to use the reference variation for the equivalence limit only when $\sigma_{g}^{2}:\sigma_{e}^{2}$ is much greater than zero, say $\sigma_{g}^{2}:\sigma_{e}^{2}>0.5$. When $\sigma_{g}^{2}:\sigma_{e}^{2}$ is small, the equivalence limit should consider other sources of variation, such as those discussed by [14].

Clearly, the conditional mean will need a different equivalence criterion because of its independence from the background effect. A plausible equivalence criterion for the conditional mean likely should consider the following: residual variation–the size of the standard error of the conditional mean difference, and the reference variation–providing biological context [15]. Ideally, such a criterion should be predetermined, like the 80–125% rule [16] used in the regulation of the generic drugs, except that the criterion for a GM crop would be on the variation instead of mean level when sufficient reference variation exists. The criterion should be general enough for all types of characteristics in terms of the variation setting $\sigma_{g}^{2}:\sigma_{e}^{2}$. In addition, the criterion should be able to take account of a GM trait-by-site interaction, when needed. These questions are topics of ongoing research.

## Conclusion

The equivalence testing method prescribed by [2] directly compares a GM crop with commercial references, and confounds the so-called GM trait effect with genotypic differences between the reference materials and control. Critically, these genotypic differences are the result of conventional plant breeding. Thus, the result of EFSA equivalence testing often has little or nothing to do with the GM trait effect, which should be the sole focus of the comparative assessment. A novel statistical model, called the trait model, was introduced that treats the effects of the GM and control materials as fixed for their difference, and as random for their common background. For significance testing, the covariance structure of the three genotype groups is utilized to decompose the differences into the trait effect and the control background effect. The trait difference is then derived as a conditional mean, assuming a background effect equal to zero; this is equivalent to statistically adjusting the control background to the reference mean. Consequently, the comparative assessment is independent of the natural genotypic differences between the control and references, which substantially reduces both Type I and Type II errors and corrects a fundamental flaw in the testing method mandated by the GMO Panel of the European Food Safety Authority.

## Supporting information

S1 File Raw data in EFSA example. (XLSX)
